# Case Report: Preputial reconstruction in a dog using bilateral caudal superficial epigastric axial pattern flap and internal lamina of prepuce

**DOI:** 10.3389/fvets.2025.1613411

**Published:** 2025-08-19

**Authors:** Wongsuda Yala, Pinprakrom Chaikongkiat, Waralee Khonggrapan, Pornphan Pumpuang, Wanchart Yippaditr

**Affiliations:** Kasetsart University Veterinary Teaching Hospital Hua Hin, Faculty of Veterinary Medicine, Kasetsart University, Prachuap Khiri Khan, Thailand

**Keywords:** caudal superficial epigastric, dog, internal lamina, prepuce flap, preputial reconstruction

## Abstract

A 3.1 kg, 5-year-old male pomeranian presented with a tumor measuring 4.5 × 3 cm on the cranioventral aspect of the prepuce. On gross examination, the tumor did not appear to involve the internal lamina of the prepuce. Surgical intervention was performed using a bilateral caudal superficial epigastric axial pattern flap in combination with the internal preputial lamina following wide tumor resection Follow-up at 10 days and 6 months postoperatively showed the axial pattern flaps had healed uneventfully, with no evidence of paraphimosis and no recurrence of the tumor based on a clinical examination. The dog could urinate without evidence of discomfort and the urine flow was a steady stream without preputial urine pooling, resembling physiological urination. This technique offers a practical and broadly applicable surgical option for managing extensive preputial defects, particularly in small-breed dogs or in cases with limited local tissue availability.

## Introduction

The prepuce is a retractable tubular skin sheath attached to the ventral abdominal wall. It contains connective tissue and smooth muscle which covers, moistens, and protects the non-erectile penis. The preputial blood supply derives from the dorsal artery of the penis, cranial and caudal superficial epigastric (CSE) (1). Surgical procedures to remove part or the entire prepuce are required in cases of traumatic injury, infection, or neoplasia. Any defects resulting from the surgical removal of the cranial preputial protrusion can usually be closed primarily; however, when large portions of the prepuce are resected, extensive preputial defects can lead to chronic penile protrusion. This causes desiccation and inflammation of the penile surface and resulting in self-trauma, penile ulceration, hemorrhage, necrosis, and balanoposthitis (2, 3). Thus, a cosmetic and functional preputial reconstruction may be difficult to achieve. In cases of extensive preputial tissue loss or resection, penile amputation and scrotal or perineal urethrostomy are often required (4, 5). Various surgical interventions have been reported for the reconstruction of large resected parts of the prepuce. Several techniques have been employed in the reconstruction in single-stage, two-stage, and multi stage procedures, such as the use of a reverse axial pattern flap, a CSE axial pattern flap combined with a full-thickness buccal mucosa graft; and a bi-pedicle subdermal plexus flap combined with a free buccal mucosal graft (6–8). Nevertheless, these techniques may lead to some complications, including an increased risk associated with multiple surgical procedures, localized full-thickness necrosis of the flap, evidence of paraphimosis, and failure of a full-thickness oral graft. This report describes and assesses the results of a single-stage surgical technique with a bilateral CSE axial pattern flap combined with the internal lamina for preputial reconstruction in a male dog which can restoration of both anatomical and functional integrity of the prepuce, following wide tumor resection on the cranioventral aspect of the prepuce. The combination of axial pattern flaps and mucosal preservation offers a reliable and adaptable solution for extensive preputial or genital soft tissue defects, particularly in small-breed dogs and in situations where conventional local flap options are insufficient.

This study was performed in accordance with the Institutional Animal Care and Use Committee of Kasetsart University, Bangkok, Thailand. Informed, written consent was obtained from the dog’s owner prior to involvement in the study.

## Case description

**Figure 1:**
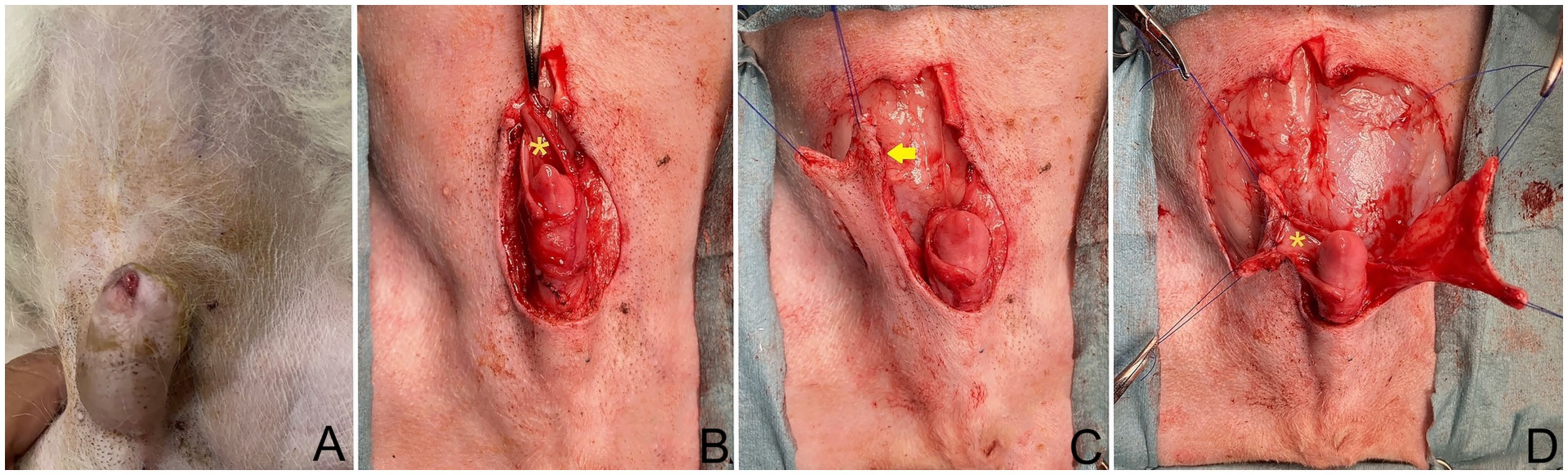
**(A)** Dog positioned in dorsal recumbency; head oriented at top of image. The tumor on the cranioventral aspect of the prepuce. **(B)** Post excision of prepuce, showing preservation of dorsal part of internal lamina (*) during tumor excision. **(C)** Full-thickness caudal epigastric longitudinal incision made along lateral internal lamina lining (arrow). **(D)** Sutured internal lamina (*) to wide and long edges of both flaps.

A 3.1 kg, 5-year-old male pomeranian was referred to the Kasetsart University Veterinary Teaching Hospital Hua Hin, Thailand, with a tumor on the cranioventral aspect of the prepuce (Figure 1A). Based on the existing information of past treatment, the dog had been treated using antibiotics and anti-inflammatories for 3 weeks at the local veterinary clinic without any improvement. Physical examination revealed a 4.5 × 3 cm tumor located on the cranioventral aspect of the prepuce. There was no sign of urinary bladder distension from abdominal palpation and there was no difficulty or discomfort during urination. The results from a complete blood count, biochemistry panel and urine analysis were unremarkable. Abdominal ultrasonography and thoracic radiographs were normal. A wide surgical excision of the tumor was performed, followed by preputial reconstruction.

Prior to the surgery, morphine at 0.2 mg/kg subcutaneously (SC), alfaxalone (Alfaxan; Jurox Pty. Ltd.) at 2 mg/kg intravenously (IV), and cephalexin (Cefaben; L.B.S. Laboratory Ltd.) at 22 mg/kg IV were administered for pain control, anesthetic induction, and as a prophylaxis antibiotic, respectively. Isoflurane and oxygen on a circular system following endotracheal intubation were used to maintain anesthesia. A constant-rate infusion of fentanyl (10 μg/kg/h) was also administered throughout surgery. The patient was placed in dorsal recumbency, with the hindlimbs extended. The prepuce was prepared aseptically by lavaging with 0.12% chlorhexidine solution (C–20; OSOTH Inter Laboratories Co., Ltd.). The tumor did not appear to involve the internal lamina of the prepuce and it was felt that there was adequate skin on the lateral aspects of the tumor for a wide excision and that the internal lamina would serve as the deep margin for excision.

The tumor on the prepuce was removed via an elliptical incision in the area of the os penis extending toward the preputial ostium, with a total length of 3.5 cm. and at the lateral aspect the incision was 1.5 cm from the edge of the tumor on prepuce. The subcutaneous tissues were divided until the external rectus sheath was reached. Then, the soft tissues attaching the prepuce to the ventral body wall were undermined, a tumor on the cranioventral aspect of the prepuce was incised 360° around the penis, with the preputial mucosa being preserved during the tumor excision (Figure 1B). Surgical gloves and instruments were changed for sterilized ones before the preputial reconstruction.

**Figure 2:**
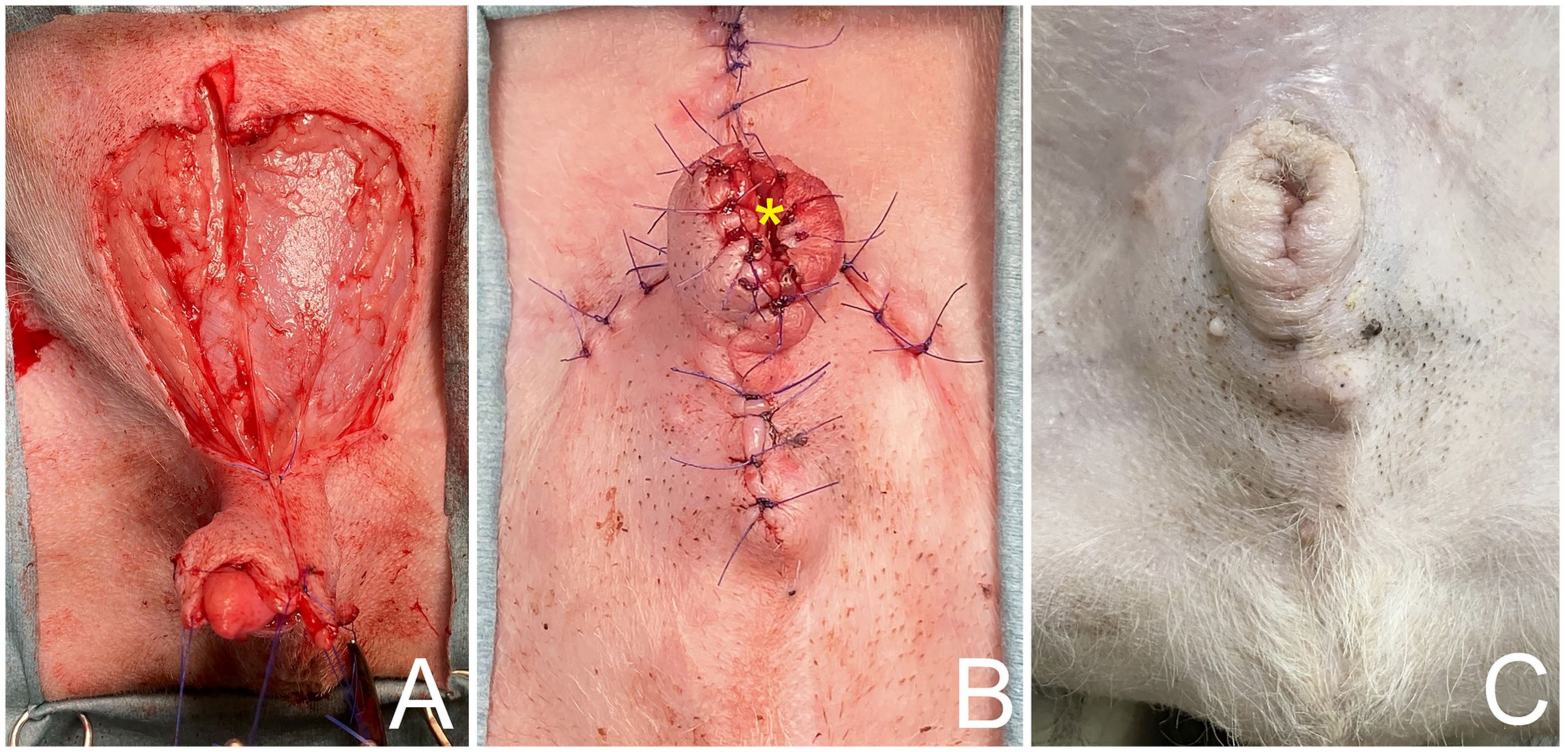
**(A)** Caudal superficial epigastric axial pattern flaps rotated to cover preputial defect and body of penis, using a simple continuous pattern with 4/0 polydioxanone subcutaneously along longitudinal edges of caudal superficial epigastric flaps. **(B)** Tubular reconstruction of prepuce and orifice (*) using axial pattern flap. **(C)** Follow-up 6 month after surgery, showing axial pattern flaps have healed uneventfully, with no visible sign of necrosis and no evidence of paraphimosis.

**Figure 3:**
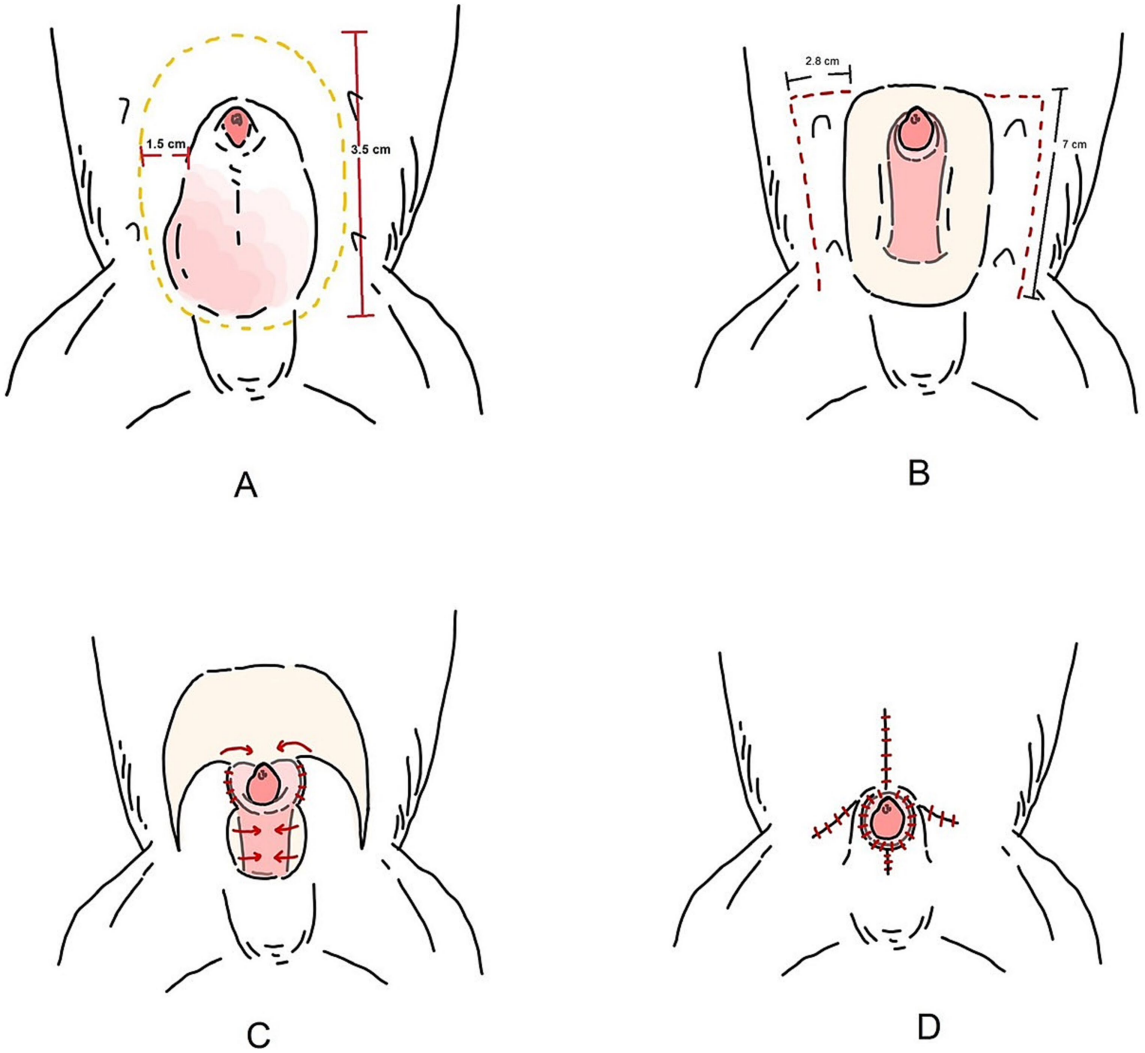
Schematic illustration of the bilateral caudal superficial epigastric axial pattern flap. **(A)** Preputial tumor excised via a 3.5 cm, elliptical incision over the os penis, with 1.5 cm lateral margin from the tumor edge. **(B)** A full-thickness longitudinal incision was made along the lateral preputial mucosa in the caudal epigastric region. **(C)** The caudal superficial epigastric axial pattern flaps were rotated to cover the preputial defect and body of the penis. **(D)** Tubular reconstruction of prepuce and orifice.

The caudal epigastric was outlined to create the flap on the right and left sides. In this case, full-thickness incisions were made along the lateral preputial mucosa lining with 2.8 cm wide and 7 cm long. Stay sutures with 4/0 polyamide were used with both the CSE axial pattern flaps and the undermined tissue below the mammary gland tissue (4th–5th pairs of mammary glands) above the external abdominal oblique muscles, taking special care to preserve most CSE branches during the undermining (Figure 1C). The CSE axial pattern flaps were rotated to cover the preputial defect and body of the penis. Pulling the internal lamina at the cranial aspect of penis, the internal lamina was sutured to the wide edges of both flaps with 4/0 polydioxanone (PDS; Johnson & Johnson International) using a simple interrupt suture pattern. No suture was applied between the preputial mucosa and the deep surface of the CSE axial pattern flap in order to avoid accidental damage of the CSE artery (Figure 1D). Thereafter, a simple continuous pattern with 4/0 polydioxanone was performed subcutaneously along the longitudinal edges of the CSE flaps, constructing a tubular structure to create a prepuce (Figure 2A). The skin of the tubular flap was sutured using a simple interrupted pattern with 4/0 polyamide (Dafilon, B. Braun Surgical). The subcutaneous tissues and skin of the donor site were closed routinely in an interrupted pattern with 4/0 polydioxanone and 4/0 polyamide, respectively (Figure 2B). Then, the penis was exteriorized to ensure it was not restricted by the newly created prepuce. The schematic illustration is shown in Figure 3.

**Figure 4:**
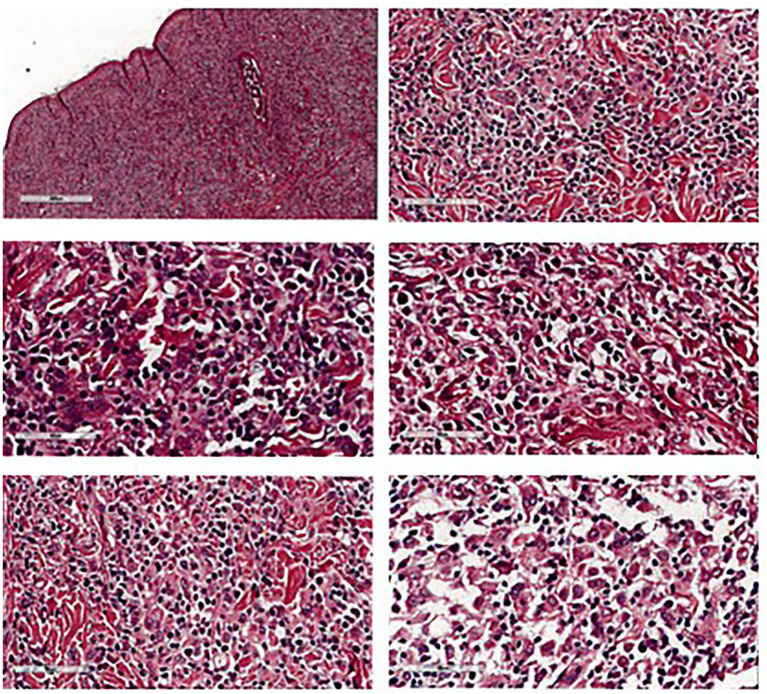
Histopathological features of preputial skin mass showed round cell tumor suggestive for histiocytoma.

Postoperatively, the dog recovered well. A 6-Fr indwelling catheter (Foley catheter; Well Lead Med. Co., Ltd.) was inserted for 5 days to avoid urinary irritation to the preputial flap and an Elizabethan collar was used to protect the surgical region from self-trauma. Amoxicillin-clavulanic acid (Amoxclamed; BIC Chemical Co., Ltd.) at 15 mg/kg was administered orally every 12 h. Morphine at 0.3 mg/kg SC was administered every 4–6 h for 3 days consecutively and carprofen (Rimadyl; Zoetis [Thailand] Ltd.) at 4.4 mg/kg was administered orally every 24 h for the following 3 days. This was for pain control. Following the removal of the urine catheter, spontaneous urination and urinary bladder distension were monitored by observing signs of tenesmus or urination on bedding, palpation of the urinary bladder. The dog was observed to urinate without evidence of discomfort and produced a steady stream of urine. At 10 days, the skin sutures were removed, as the axial pattern flaps demonstrated good adherence in both the skin and mucosal regions of the prepuce with no visible sign of necrosis. The biopsy result was histiocytoma (Figure 4). For 6 months after surgery, the surgical wound showed good healing. While the axial flap experienced slight contraction with no evidence of paraphimosis upon clinical examination (Figure 2C) and the dog was able to urinate normally without straining. The dog was followed up to assess for complications, difficulty, or discomfort. The dog had no signs of tumor recurrence or urinary problems.

## Discussion

Surgical techniques for the reconstruction of large preputial defects are limited and challenging because good cosmetic and functional outcomes are difficult to achieve. A penile amputation combined with scrotal urethrostomy is commonly chosen as the standard procedure (2, 4, 9, 10). However, a notable disadvantage is that the owner was reluctant to agree to penile amputation and scrotal urethrostomy. Reconstructive techniques have been published for extensive preputial defects. However, such techniques have reported disadvantages as mentioned above. The present study involved a novel surgical procedure to reconstruct large preputial defects to create the proximal prepuce and preputial orifice in a male dog, consisting of a bilateral CSE axial pattern with an internal laminal flap. The clinical findings identified no postoperative complications. The CSE axial pattern flap technique has been largely employed in veterinary reconstructive surgery, and has shown promising results (11–13). The caudal superficial epigastric artery and vein arise from the external pudendal artery and vein and guarantee an adequate vascular supply to the flap. The CSE axial pattern flap usually requires elevation and transposition of the last three mammary glands in dogs (1). In the dog in the current case study, the last two glands were sufficient to cover the preputial defects and allowed adequate vascularization of the whole transposed flap, which corresponded to another report where the last two glands provided adequate vascularization of the whole transposed flap (6). In addition, the flaps were easily transposed and enrolled with the apex and body of the penis. The dog recovered without any wound healing complications or signs of long-term discomfort. The flap was created using a larger preputial orifice and a longer preputial reconstruction to prevent paraphimosis and preputial orifice stenosis due to tissue contraction (30–40%) during the healing process (6, 14), which is a common complication in reported preputial reconstruction (6). Therefore, in the present case, the length and width of the flap were made greater than the preputial defect by 40%, where the width of the flap was measured based on the normal circumference of the prepuce. The mucosa from the cranial extent of the defect provided a well-vascularized flap with the blood supply derived from the external pudendal artery and veins (2, 15). The preserved internal lamina was used as an advancement flap to create the new preputial tissue and preputial orifice, to guide urine flow and minimize scalding. This was simpler than the use of oral mucosal grafts (6, 8). The creation of a tubular structure for the prepuce closely resembled the normal physiology, with the penis in a normal location and not attached to the dorsal body wall without placing tension on the flap or penis (7). A limitation of this report is that ultrasonographic evaluation of the preputial mass itself was not performed prior to surgery. Although abdominal ultrasonography was unremarkable, direct imaging of the mass could have aided in preoperative diagnostic assessment and surgical planning.

In summary, the bilateral CSE axial pattern with an internal laminal flap was a successful surgical procedure to create a prepuce and preputial orifice. This technique could be applied for the simple, one-stage reconstruction of large preputial defects following tumor excision or to treat extensive traumatic preputial damage not involving the internal lamina of the prepuce, allowing for the preservation of the penis, with the new preputial structure closely resembling a physiological prepuce. However, this technique might not be suitable for cases with extensive skin tissue damage around the 4th–5th pairs of mammary glands, or for cases where the tumor in the prepuce deeply invades the internal lamina of the prepuce. Overall, our study should help in mitigating complications and in promoting better patient management.

## Data Availability

The raw data supporting the conclusions of this article will be made available by the authors, without undue reservation.
