# Supplementary figures and images for: Case Report: Preputial reconstruction in a dog using bilateral caudal superficial epigastric axial pattern flap and internal lamina of prepuce
Source: Front Vet Sci. 2025 Aug 19;12:1613411. doi: 10.3389/fvets.2025.1613411 (PMC12401979; doi:10.3389/fvets.2025.1613411)

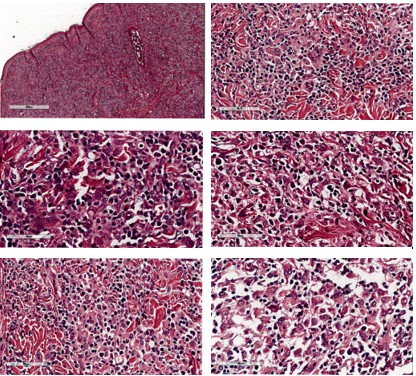

Supplement: Supplementary file 1 [file Image_1.jpeg]
